# Neuroinflammation and neurohormesis in the pathogenesis of Alzheimer’s disease and Alzheimer-linked pathologies: modulation by nutritional mushrooms

**DOI:** 10.1186/s12979-017-0108-1

**Published:** 2018-02-14

**Authors:** Angela Trovato Salinaro, Manuela Pennisi, Rosanna Di Paola, Maria Scuto, Rosalia Crupi, Maria Teresa Cambria, Maria Laura Ontario, Mario Tomasello, Maurizio Uva, Luigi Maiolino, Edward J. Calabrese, Salvatore Cuzzocrea, Vittorio Calabrese

**Affiliations:** 1 Department of Biomedical and Biotechnological Sciences, School of Medicine, University of Catania, Via Santa Sofia 97, 95123 Catania, Italy; 2 Department of Chemical, Biological, Pharmaceutical and Environmental Sciences University of Messina, Messina, Italy; 3 Department of Medical and Surgery Sciences and Advanced Technology, University of Catania, Catania, Italy; 4 Spinal Unit, Emergency Hospital “Cannizzaro”, Catania, Italy; 5 Environmental Health Sciences Division, School of Public Health, University of Massachusetts, Amherst, MA USA

**Keywords:** Oxidative stress, Neurodegenerative disorders, Neurohormesis, Mushrooms

## Abstract

Human life develops and expands not only in time and space, but also in the retrograde permanent recollection and interweaving of memories. Therefore, individual human identity depends fully on a proper access to the autobiographical memory. Such access is hindered or lost under pathological conditions such as Alzheimer’s disease, including recently associated oxidant pathologies, such as ocular neural degeneration occurring in glaucoma or neurosensorial degeneration occurring in Menière’s disease. Oxidative stress and altered antioxidant systems have been suggested to play a role in the aetiology of major neurodegenerative disorders, and altered expression of genes sensing oxidative stress, as well as decreased cellular stress response mechanisms could synergistically contribute to the course of these oxidant disorders. Thus, the theory that low levels of stress can produce protective responses against the pathogenic processes is a frontier area of neurobiological research focal to understanding and developing therapeutic approaches to neurodegenerative disorders. Herein, we discuss cellular mechanisms underlying AD neuroinflammatory pathogenesis that are contributory to Alzheimer’s disease. We describe endogenous cellular defence mechanism modulation and neurohormesis as a potentially innovative approach to therapeutics for AD and other neurodegenerative conditions that are associated with mitochondrial dysfunction and neuroinflammation. Particularly, we consider the emerging role of the inflammasome as an important component of the neuroprotective network, as well as the importance of *Coriolus* and *Hericium* nutritional mushrooms in redox stress responsive mechanisms and neuroprotection.

## Background

Alzheimer’s disease (AD) is a neurodegenerative progressive disorder affecting more than 15 million people worldwide and represents the most current cause of dementia in the elderly, accounting for 50–60% of all cases in Western world [1].

The pathological signs of AD are amyloid plaques containing amyloid-β (Aβ) peptide derived from trans-amyloid precursor protein and neurofibrillary tangles constituted by hyper phosphorylated tau protein in medial temporal lobe structures and cortical areas of the brain along with neuronal death and synapse loss [2]. It has been demonstrated that inflammation cascade is linked to neurodegenerative diseases, particularly, Alzheimer’s disease (AD) [3, 4]. In order to resist to different injuries, brain cells have developed networks of responses that detect and control different forms of stress [5, 6]. These are mainly proteins, including heat shock proteins (Hsps), lipoxin A4 (LXA4), thioredoxin (Trx) and sirtuins, controlled by vitagenes, a redox-dependent complex of genes [7]. LXA4 is an endogenous eicosanoid, produced by arachidonic acid metabolism, endowed with anti-inflammatory properties in different inflammatory syndromes, such as periodontitis, nephritis, arthritis, inflammatory bowel disease. LXA4 blocks the productions of pro-inflammatory mediators including free radical oxygen and nitrogen reactive intermediates (ROS/RNS), and acts as an endogenous “braking signal” in the inflammatory process. It is generally acknowledged as “signal stop” of inflammation [8]. The discovery of agents capable of increasing LXA4 levels and subsequently Aβ uptake by phagocytic cells is increasingly being recognized as a potential therapeutic target for AD treatment. Previous studies have demonstrated that mushrooms significantly up-regulate LXA4 in the brain. Mushrooms have been used in traditional medicine, for many years [9], and a long list of therapeutic properties exists that have been associated with mushroom extracts, including antitumor, immunomodulatory, antioxidant, antiviral, antibacterial, and hepatoprotective effects. Mushrooms are a rich font of polysaccharides, and many of them have been shown to stimulate host immune responses. Among the most powerful known immunostimulators, β-glucans derived from mushrooms stimulate immune cells and cytokine responses [8, 10].

Taken into account the fact that Alzheimer’s disease is characterized by neurodegeneration associated with neuroinflammation, in our recent study we have shown that *Coriolus versicolor* and *Hericium erinaceus* biomass preparations have neuroprotective effects and act by modulating the inflammatory process associated with the pathology of AD, as well as regulating brain cellular stress response mechanisms [4].

There is a growing body of evidence demonstrating a link between Alzheimer’s disease and glaucoma. Notably, amyloid deposits, constituted of amyloid beta (Αβ) aggregates, a characteristic feature of several neurodegenerative diseases, such as Alzheimer’s, mild cognitive impairment and Parkinson’s disease (PD), have been recently implicated in the pathogenesis of retinal damage, of age-related macular degeneration and glaucoma. Glaucoma is a progressive optic neuropathy characterized by gradual degeneration of neuronal tissue due to retinal ganglion cell loss, associated to visual field loss over time resulting in irreversible blindness [11, 12]. It is a leading cause of irreversible blindness estimated to affect 79.6 million people worldwide by 2020. Accumulation of Aβ characterizes glaucoma as a protein misfolding disease, suggesting a pathogenic role for oxidative stress in the pathogenesis of retinal degenerative damage associated to this ocular pathology. In particular, factors such as tissue hypoxia and disturbed protein metabolism have been identified to interact in a vicious cycle underlying the oxidative stress-driven pathogenesis of glaucoma, ultimately leading to apoptotic retina ganglion cell death [13]. Research studies have demonstrated that retinal ganglion cell (RGC) damage in glaucoma is not limited to the primary insulted neurons, but also involves neighbouring neurons. In view of these considerations glaucoma can be viewed as a neurodegenerative disease which, similarly to other neurodegenerative pathologies, i.e., Alzheimer’s and Parkinson’s disease, where irreversible functional deficit ensues as consequence of neuronal dysfunction and death. Interestingly, recent evidence from our laboratory have demonstrated higher levels of vitagenes Heat Shock Protein 72 (Hsp72) and Heme oxygenase (HO-1) in the blood of patients with glaucoma than in controls [14]. These changes were associated with an increased expression of Trx and sirtuin 1 in the same experimental group. Similar results have been found in another oxidant disorder impacted by a progressive degenerative damage of neurosensorial acustic system, such as Ménière’s disease [15]. Ménière’s disease (MD) is characterized by the triad of fluctuating hearing loss, episodic vertigo and tinnitus, and by endolymphatic hydrops found on post-mortem examination. Increasing evidence suggests that oxidative stress is involved in the development of endolymphatic hydrops and that cellular damage and apoptotic cell death might contribute to the sensorineural hearing loss found in later stages of MD [16]. Consistent with this notion, studies are presently under way in our laboratory testing the conceivable possibility that mushrooms supplementation can reverse oxidative damage, thereby affecting the clinical course of Meniere’s disease pathology. Thus, modulation of endogenous cellular defense mechanisms such as the vitagene network may open to new therapeutic approaches in diseases associated with tissue damage and cell death, such as in glaucomatous or Meniere neurodegeneration [14, 15]. In this review, we specifically discuss the main neuroprotective and nutritional activities of *Coriolus* and *Hericium* mushrooms. Moreover, we will introduce the emerging role of hormesis and inflammasome as important components of neuroprotective network operating in redox-dependent brain cell stress responsive mechanisms. Our focus presently highlights the hypothesis linking oxidative stress and neurodegeneration to the AD pathogenesis, and indicate that stress responsive genes may represent an important target for novel cytoprotective strategies, as molecules inducing this defense mechanism, via nutritional and/or pharmacological approaches, can exploit the potential for antidegenerative therapeutic interventions [14].

### Neurohormesis

At the core of adaptive responses at the cell and origin of biological organization is the concept of hormesis [17]. Hormesis is the expression of integrative adaptive responses that are manifest via a biphasic dose response with very specific quantitative response feature (i.e. maximum amplitude and width of the adaptive response) and induced by either a direct stimulatory response or as a modest overcompensation to a disruption of homeostasis [18]. Detailed assessments of pre- and post-conditioning, the adaptive response in radiation, the priming response, as commonly reported in microbial and plant models, and the so-called steeling effect widely reported in the clinical psychology area, all these phenomena act via hormetic mechanisms and comprise a broadly integrated and evolutionarily based adaptive system that has been highly conserved [19]. Such hormetic dose responses provide a quantitative description of the bounds of biological plasticity [20], and a measure of the extent to which adaptive processes may be upregulated, which is especially relevant to the comprehension of protective effects induced by plant and fungal species. The hormetic concept is particularly important since it provides reliable estimates of the upper limit for the induction of potential therapeutic responses and should play a key role in the design of experimental studies and clinical trials. Hormesis, expecially in vulnerable biological systems, such as the brain, is of relevant interest to the toxicological community for the dose-response model. Particularly, neurohormesis affects memory, learning and performance, as well as nutritional antioxidants and neurodegenerative responses mediated by oxidative stress in cellular models for various diseases such as AD [9, 17]. In fact, the presence of oxidative stress markers in the brains of patients with neurodegenerative diseases has been recognized which supports the rationale for neuroprotective nutritional interventions based on the action antioxidants and anti-inflammatory agents, such as polyphenols or mushrooms [7]. Neurohormesis can be applied to both polyphenol and nutritional mushroom molecular mechanisms of action. It has been, in fact, known that polyphenols and mushrooms activate the heat shock protein (Hsp) pathway, which plays a crucial role in the cellular stress response. With respect to this, for instance although there is no doubt about the protective effect of HO-1 against oxidative and nitrosative stress, on the contrary, excessive HO-1 upregulation may be toxic for cells. According to this principle, drugs, toxins and natural substances, administered at low doses can result in a positive response, while at higher concentrations promote prevalent toxic effects [17].

### Role of the inflammasome

**Fig. 1:**
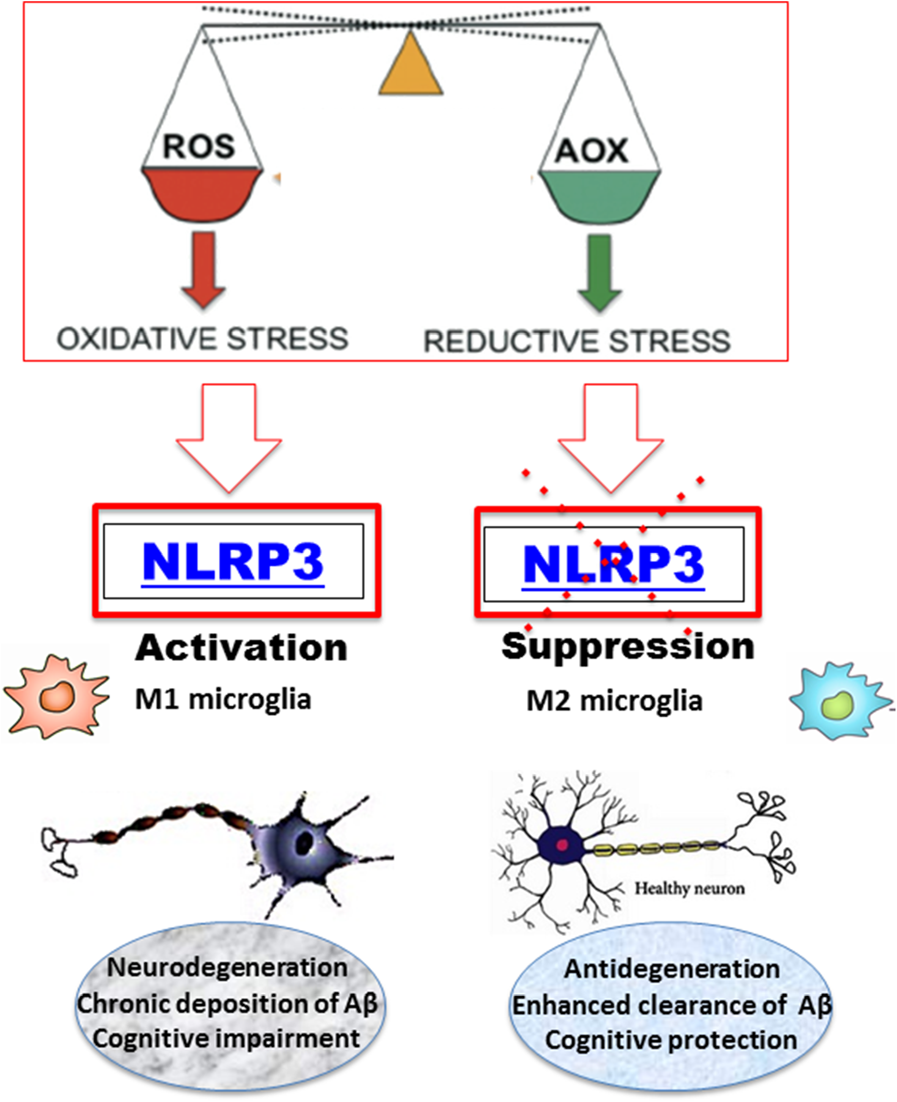
NLRP3 and redox alterations in the pathophysiology of Alzheimer disease: Redox equilibrium is maintained by the balance of ROS and antioxidants. If ROS prevail, cells undergo oxidative stress and NLRP3 inflammasome activation. If antioxidants prevail, reductive stress may occur with NLRP3 inflammasome suppression

Oxidative stress is one of the main components of the pathogenesis of neurodegenerative diseases. The molecular mechanisms underlying oxidative stress include inflammation, mitochondrial dysfunction and apoptosis that culminates in neuronal death [21, 22]. Modulation of cellular stress pathways through the use of small redox active molecules represents a new approach to the study of neurologic and psychiatric pathologies [23]. In particular sulforaphane and hydroxytyrosol, as well as nutritional mushrooms are increasingly considered as possible candidates to regulate physiological pathways related with (1) cellular stress response and vitagene networks; (2) redox imbalance/oxidative stress, (3) mitochondrial function (4) immune response and anti-neuroinflammation (5) heat shock response control, and (6) synaptic dysfunction [24]. Excessive oxidative stress levels have been correlated to AD pathogenesis, increased protein carbonylation, nitration, cysteine-oxidation, lipid peroxidation, and DNA/RNA oxidation, were revealed in brain and peripheral tissue samples from patients with AD by post-mortem studies. Increased oxidative stress can damage mitochondrial proteins. Of particular significance to the pathophysiology of main neurodegenerative disorders are findings of reduced levels of mRNA (and protein subunits) that are involved in the transfer of electrons in complex I of the electron transport chain (ETC), in AD patients. Decreased efficiency of the electron transfer process within complex I and complex IV, results in increased leakage and mono-electronic reduction of molecular oxygen to form the superoxide anion [25], with ensuing damage to proteins, lipids and DNA. As well, increasing evidence supports a role of immune activation as a prominent causative factor in the pathogenesis of a number of major neurologic and neuropsychiatric disorders [26]. Consistent with this notion, current studies have demonstrated that the inflammasome modulates neuroinflammatory processes at the initial stage, with a secondary cascade of events inclusive of oxidative stress, redox homeostasis disruption associated to mitochondrial dysfunction (Fig. 1) [27]. The inflammasome is a multiprotein complex that contains many copies of a receptor for pathogen- or damage-derived molecular patterns (Pathogen Associated Molecular Patterns, PAMPs), pro-caspases-1, and an adaptor, apoptotic speck-containing protein with a caspase activation and recruitment domain (CARD) [ASC], which induces caspase-1 maturation [28]. Active caspase-1 is responsible for rapid, lytic cell death (pyroptosis). Upon sensing PAMP or damage associated molecular pattern (DAMP), absent in melanoma 2 (AIM2) and/or PYD domains-containing protein 3 (NLRP3), inflammasomes activate caspase-8 and caspase-1, respectively, leading to both pyroptotic and apoptotic cell death [29]. Mitochondria represent major sources of DAMPs capable of triggering neuroinflammatory responses, with resulting pyroptosis, apoptosis and autophagy [30]. AIM2 is a cytoplasmic sensor that recognizes and binds the double-stranded DNA (dsDNA) of microbial or host origin. Upon binding to DNA, AIM2 assembles inflammasome complex, which induces pyroptosis and proteolytic cleavage of the proinflammatory cytokines pro-IL-1β and pro-IL-18. A wrong recognition of cytoplasmic self-DNA by AIM2 provides to the development of autoimmune and inflammatory diseases, as well as neurodegenerative disorders [31].

A direct activation link between inflammation and the pathogenesis of Alzheimer’s disease has been demonstrated by numerous in vitro and in vivo studies. in particular the inflammasome NLRP £ has been identified as a possible therapeutic target for the treatment of neurodegenerative diseases. This hypothesis was accredited by the observation of a deficiency of NLRP3 and caspase-1 in mice AA / PS1 (transgenic mice for chronic deposition of Ab), in which Ab deposits were reduced, resulting in an increase in M2 microglia [32]. We have provided recent evidence of a neuroprotective action of the *Hericium erinaceus (*MRLs, UK) mushroom when administered orally to rat. In the brain of rats receiving oral administration of *Hericium erinaceus*, was measured maximum expression of LXA4, an anti-inflammatory compound, in cortex and hippocampus. LXA4 up-regulation was related with an increased amount of proteins, such as thioredoxin, Hsp72 and heme oxygenase-1 involved in cellular stress response [4].

Plausibly, LXA4 signalling activation and stress-responsive vitagene proteins modulation could serve as a potential therapeutic target for AD-related inflammation and neurodegenerative damage. Our results indicate that nutritional supplementation with an opportune biomass preparation from a well characterized strain of *Hericium erinaceus* or *Coriolus versicolor* can induced critical proteins modulation involved in brain age-associated neurodegenerative diseases [33]. In view of the fact that in AD pathology, amyloid plaques accumulation (APs), composed of amyloid-beta peptide (Aβ) aggregates, and neurofibrillary tangles (NFTs) formation, composed of misfolded Tau proteins, are related with a deficit in those mechanisms participating in the induction of cytoprotective proteins (Hsps) or, more generally, cellular pathways of stress tolerance, it is conceivable to hypothesize that in these conditions, administration of *Hericium erinaceus* or *Coriolus versicolor* biomass, by enhancing the redox potential and inducing neuroprotection through neurohormetic mechanisms such as vitagenes upregulation, may promote resilience in damaged neurons, and hence resistance to proteotoxic insults and apoptotic neurodegeneration. Consistent with this concept, restoration of normal proteostasis is crucial for neuronal survival. Our research suggests new potential strategies based on the induction of vitagene defence system as a foundamental mechanism to promote proteome homeostasis and hence withstand pathological mechanisms associated to unhealthy aging of the brain associated to neurodegenerative diseases [4].

#### *Coriolus versicolor*

**Fig. 2:**
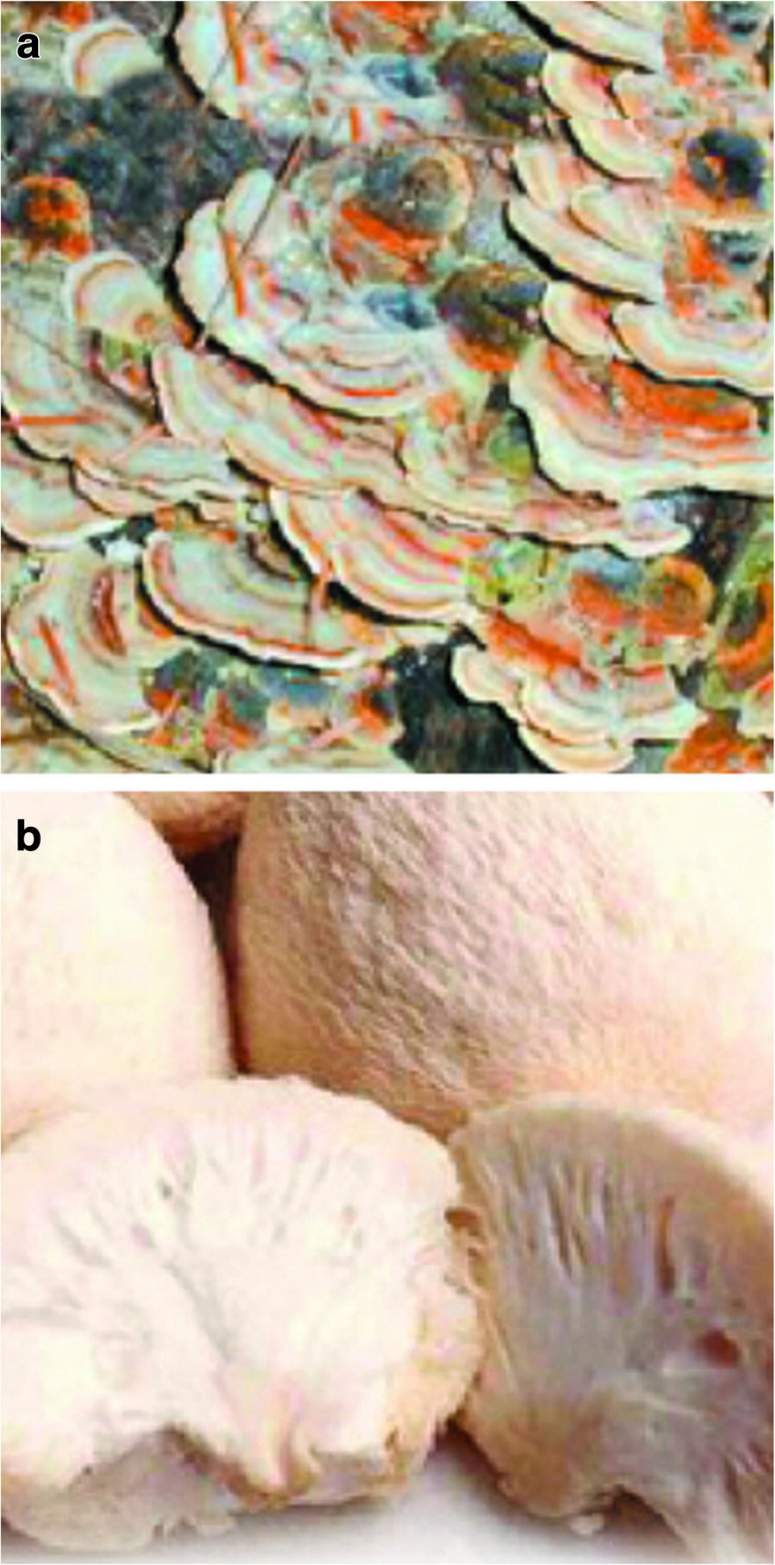
*Coriolus versicolor* (**a**) and *Hericium erinaceus* (**b**) mushrooms

The medicinal properties of mushrooms have long been known to traditional medicine (Fig. 2a, b) [34, 35]. Anti-oxidant, anti-bacterial, and anti-viral properties have been attributed to mushrooms by controlled studies [36]. It has been shown that mushrooms are capable to stimulate the immune system of the host due to the high content of β-glucans, which activate many types of immune cells and stimulate cytokine responses [37–39]. Several of these polysaccharides are currently used in East countries in association to radio and chemotherapy [40]. In addition, Cordymin, a peptide with low molecular weight (10,906 Da), with anti-inflammatory properties has been isolated from the medical mushroom *Cordyceps sinensis* [41] and from *Cordyceps militaris* [42]. This peptide significantly inhibited the polymorphonuclear cells infiltration and IR-induced up regulation of C3 protein produced in the brain, interleukin-1β, and tumour necrosis factor-α, which had a neuroprotective effect on the ischemic brain, due to the inhibition of inflammation [34].

Although the polysaccharides derived from mushrooms are hardly synthesizable molecules and the active molecules present in the mushrooms are still not well known, the Asian clinical practice employs preparations derived from mushrooms, including *Agaricus campestris*, *Pleurotus ostreatus* and *Coriolus versicolor* extracts [43]. Polysaccharides obtained from *coriolus versicolor* (Fig. 2a) are commercially among the best options. This mushroom is known for its medical applications, and is usually used to degrade organic contaminants such as pentachlorophenol (PCP) [44]. Polysaccharide K or Kerstin (PSK) and polysaccharopeptide (PSP) are the main commercial preparations obtained by C *versicolor* mycelia [45]. These compounds stimulate the production of interleukin-6, interferons, immunoglobulin G, macrophages, and T-lymphocytes by enhancing the immune system against immunosuppressive effects of radiotherapy and chemotherapy. Polysaccharopeptides possess anticancer activity inhibiting the production of superoxide dismutase (SOD) and glutathione peroxidase [46]. Experimental animal and human studies have shown that oral administration of PSK and PSP controlled carcinomas [47]. It is reported that PSK produces apoptosis in HL-60 Human promyelocytic leukaemia cells through the activation of mitochondrial and caspase-dependent pathways [48] and overexpression of proapoptotic protein Bax [49]. Of particular interest are the polysaccaropeptides produced by *Coriolus versicolor, which* are used to supplement the chemotherapy and radiotherapy of cancer and infectious diseases. Chronic inflammation favours the progression of Alzheimer’s disease (AD), however identification of mechanisms able of resolving the pro-inflammatory environment stimulating AD pathology remains an area of active investigation [50, 51]. Taking into account this neurobiological rationale, our recent study, carried out to demonstrate a potential neuroprotective role of coriolus versicolor biomass preparation in neuroinflammatory pathogenesis associated with Alzheimer’s disease in rat, has been undertaken to provide experimental evidence that biomass from *Coriolus* or *Hericium* regulate important redox sensitive pathways linked to cellular stress response and hence confer neuroprotection [3, 4, 52]. Lipoxin A4 (LXA4) derived from *Coriolus* is an endogenous eicosanoid capable to resolving inflammation process, acting as an endogenous “braking signal” in the inflammatory cascade. Treatment with the pro-resolving mediator aspirin-triggered lipoxin A4 (ATL), caused cognition improvement, reduced Ab levels, and enhanced phagocytic activity of microglia in Tg2576 transgenic AD mice [53]. Moreover, LXA4 levels declined with age, a finding even more evident in 3xTg-AD mice [54]. LXA4 action is regulated by the interaction with G protein-coupled receptor. N-formyl-peptide receptor 1 (FPRL1), also known as ALX (lipoxin A4 receptor) or CCR12, belongs to the formyl-peptide receptor (FPR)-related family of G protein-coupled receptors (GPCRs) that also includes FPR and FPRL2 [55, 56]. All factors able to increase Lipoxin A4 (LXA4) levels and consequently uptake of Ab by phagocytic cells are a hypothetical therapeutic target for AD. Consistent with this notion, in AD pathology, the accumulation of amyloid plaques (APs), constituted of amyloid-beta peptide (Ab) aggregates, and neurofibrillary tangles (NFTs), constituted of misfolded Tau proteins, is related to a deficiency in the activation of cytoprotective proteins (Hsps) or, more generally, of cellular stress tolerance pathways [55]. In these conditions, it is demonstrated that the administration of *Coriolus* in the brain of rats causes the maximum induction of LXA4 in cortex and hippocampus. Notably, no significant modifications in I-Kappa-B-Alpha (IkBa), Nuclear Factor Kappa B (NFkB) and cyclooxygenase-2 (COX-2) brain levels were associated with Hsps induction [4]*.* Furthermore, LXA4 up-regulation is related with Nrf-2 regulated vitagenes, thus increasing the content of proteins involved in cellular oxidative stress response, such as thioredoxin, Hsp72 and heme oxygenase [52]*.*Therefore the induction of vitagenes, could help vulnerable neurons resist proteotoxic insults and to reduce apoptotic neurodegeneration.

#### *Hericium erinaceus*

*Hericium erinaceus* (Fig. 2b) fruit bodies and mycelia contain an extraordinarily large quantity of structurally different bioactive and potentially bioactive components. The reported health-promoting properties of these compounds include anticarcinogenic, antibiotic, antidiabetic, antifatigue, antihypertensive, antihyperlipodemic, antisenescence, cardioprotective, nephroprotective, hepatoprotective, and neuroprotective properties and improvement of anxiety, depression and cognitive function [57]. The antioxidant activity of *Hericium erinaceus* has been observed in diabetic rat model in which the intra peritoneal (i.p.) administration of an aqueous extract of *Hericium erinaceus* (100 and 200 mg/kg body weight) resulted in a significant decrease in the serum glucose level, significant increase in the insulin level and attenuated serum lipid profiles (disorders) as compared to control rats. These findings were accompanied by increased activities in the antioxidative enzymes catalase (CAT), superoxide dismutase (SOD), and glutathione peroxidase (GSH-Px) and increased GSH (glutathione) and reduced malondialdehyde (MDA) levels in the liver, suggesting that the mechanism of the health-promoting effects seems to be the result of inhibition of ROS [58]. Endowed with different biological activities, *Hericium-*derived hericenones and erinacines, isolated from its fruiting body, stimulate nerve growth factor (NGF) synthesis in cultured astrocytes [59]. NGF influences basal forebrain cholinergic neurons modulating the activity of two enzymes, such as cholineacetyltransferase and acetyl cholinesterase. The first pathological events of Alzheimer’s disease are the loss and dysfunction of cholinergic neurons. *Hericium erinaceus* administration improves cognitive dysfunction. Less, however, is known about the clinical relevance of *Hericium erinaceus* in regulating neurogenesis in the nervous system and its role in neurodegenerative disorders such as Alzheimer’s disease and other types of dementia. Recently, basic and clinical studies have shown that Alzheimer’s disease is closely associated with amyloid beta (Aβ)-induced neuroinflammation, responsible for, the resident macrophages of the brain, and activated microglia may then promote neuronal injury through the release of proinflammatory and cytotoxic factors, exacerbating the course of the disease [60]. A new neuroprotective strategy, like an oral administration of a biomass *Hericium* biomass preparation given for 3 month, as done in our recent study [4] can represent a therapeutic target to minimize the deleterious effects related to oxidative burden, such as that occurring in brain aging and in neurodegenerative disorders. Treatment with *Hericium* e*rinaceus* caused a significant increase of LXA4 production in most of the brain regions like cortex, hippocampus followed by substantia Nigra, striatum and cerebellum and in a modulated expression of cytoprotective proteins, such as Heme oxygenase 1 (HO-1), Heat Shock Protein 70 (Hsp70) and thioredoxin (TRX). These results are coherent with recent evidence obtained in mice, showing neuroprotection by *Hericium erinaceus* on Aβ 25–35 peptide-induced cognitive dysfunction [61, 62].

## Conclusions

Accumulating evidence has indicated that oxidative stress and excess reactive oxygen and nitrogen intermediates play an important role in the progression of many chronic inflammatory diseases, including cardiovascular diseases, diabetes, and neurodegenerative disorders [63]. Imbalance between ROS generation and antioxidant enzyme activities will cause lipid peroxidation, nuclear and mitochondrial DNA damage and protein oxidation, resulting in brain damage and amnesia [64]. A growing number of studies have demonstrated that dietary interventions regulate mitochondrial ROS production, detoxification and oxidative damage repair. Many (but not all) of these nutritional interventions are related with extension of lifespan, or protection against diseases related with age, in mammals. Emerging nutraceuticals are today showing promise as modulators of mitochondrial redox metabolism capable of eliciting beneficial outcomes. Mushrooms, known for their strong antioxidant properties, have attracted interest due to their potential in neuroprotection, antioxidant, and anti-inflammatory effects, as well as in proteome and mitochondrial homeostasis restoration as a basic mechanism to withstand mitochondrial dysfunction-associated neuroinflammatory disorders.

## Abbreviations

AD: Alzheimer’s disease
AIM2: Absent in melanoma 2
ALX: Lipoxin A4 receptor
APs: Amyloid plaques
ASC: Apoptotic speck-containing protein
ATL: Aspirin triggered lipoxin A4
Aβ: Amyloid-beta peptide
CAT: Catalase
CBF: Cerebral blood flow
COX-2: Cyclooxygenase-2
*DAMP*: Damage associated molecular pattern
ETC: Electron transport chain
FPRL1: N-formyl-peptide receptor 1
GPCRs: G protein-coupled receptors
GSH: Glutathione
GSH-Px: Glutathione peroxidase
HO-1: Heme oxygenase 1
Hsp70: Heat shock protein 70
Hsp72: Heat shock protein 72
Hsps: Heat shock proteins
IkBa: I-Kappa-B-Alpha
LXA4: Lipoxin A4
MDA: Malondialdehyde
NFkB: Nuclear factor kappa B
NFTs: Neurofibrillary tangles
NGF: Nerve growth factor
NLRP3: PYD domains-containing protein 3
PCP: Pentachlorophenol
PD: Parkinson’s disease
PSK: Polysaccharide K or Krestin
PSP: Polysaccharopeptide
RGC: Retinal ganglion cell
ROS: Radical oxygen species
SOD: Superoxide dismutase
*TRX*: Thioredoxin
